# Nocardia rubra cell‐wall skeleton influences the development of cervical carcinoma by promoting the antitumor effect of macrophages and dendritic cells

**DOI:** 10.1002/cam4.4526

**Published:** 2022-01-07

**Authors:** Siyang Zhang, Han Wang, Yisi Liu, Tao Tao, Zhi Zeng, Yingying Zhou, Min Wang

**Affiliations:** ^1^ Department of Obstetrics and Gynecology Shengjing Hospital of China Medical University Shenyang China; ^2^ Department of Obstetrics and Gynecology Cancer Hospital of China Medical University Shenyang China

**Keywords:** cervical carcinoma, dendritic cell, innate immune, macrophage, Nocardia rubra cell‐wall skeleton

## Abstract

**Background:**

As an immune enhancer, Nocardia rubra cell‐wall skeleton (Nr‐CWS) has been used to treat persistent human papillomavirus infection and cervical precancerous lesions. However, it is still unclear whether it can be used to treat cervical carcinoma.

**Methods:**

In our study, the aim was to determine whether Nr‐CWS affects the apoptosis of cervical carcinoma cells by enhancing the antitumor effect of dendritic cells and macrophages in vivo and in vitro.

**Results:**

The experimental results showed that Nr‐CWS can promote the activity of dendritic cells and macrophages and reduce their apoptosis. It also increased the cytokines IL‐6, IL‐12, TNF‐ɑ, and IL‐1β secreted by dendritic cells and macrophages and reduced their PD‐L1 expression. In vitro, Nr‐CWS inhibited the proliferation, colony forming ability of HeLa and SiHa cervical carcinoma cell lines cultured with macrophages, and more cells were blocked in G2/M phase. Nr‐CWS promoted TNF‐ɑ/TNFR1/caspase‐8‐mediated apoptosis by increasing macrophages secretion of TNF‐ɑ and inhibited cell migration and invasion regulated by the WNT/β‐catenin‐EMT pathway. Nr‐CWS also reduced the expression of the cervical carcinoma genes E6 and E7 thereby increasing expression of p53 gene and decreasing expression of PD‐L1 gene. In vivo, Nr‐CWS inhibited tumor growth and decreased the expression of E6, E7, PD‐L1, P16, Ki67, and PCNA in tumors.

**Conclusions:**

Therefore, our results suggest that Nr‐CWS can promote apoptosis of cervical carcinoma cells by enhancing the antitumor effect of dendritic cells and macrophages.

## Graphical abstract

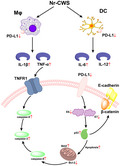

## 1 INTRODUCTION

Cervical carcinoma is a major cancer affecting women's health and one of the highest rates of cancer‐related deaths in developing countries.^1^ The pathogenesis of cervical carcinoma has been proven to be related to HPV virus infection and patient immune degradation.^2^ However, to date, no antiviral drugs that are effective in eliminating HPV have been identified. The blocking and self‐clearing of local cervical HPV infection depend on the state of the host's immune system. Therefore, immunotherapy plays a vital role in the treatment of cervical carcinoma.

Although T cells are the primary effectors of the antitumor response, there is increasing evidence that innate immune cells are essential for successful tumor clearance.^3^ Dendritic cells (DCs) have been described as sentinels of the immune system. Although DCs are only a small proportion of immune cells in tumors and lymphatic organs, these cells are central to initiating antigen‐specific immunity and tolerance. They continuously collect antigens and present them to T cells through MHC I and II,^4^ and modulate the activity of other immune cells through cell‐to‐cell contact and cytokine release, thereby shaping the immune response.^5^

Ideally, newly emerging tumor cells can be recognized and eliminated by innate immune cells such as macrophages.^6^ As the most abundant innate immune cells in tumor‐infiltrating immune cells, tumor‐associated macrophages (TAMs) are the main inflammatory cell types in the tumor microenvironment.^7^ TAMs include inactive M0 macrophages, M1 macrophages with antitumor effect, M2 macrophages with tumor‐promoting effect, and many macrophages whose classification has not been clearly defined.^8^ Secretion of IL‐12 by macrophages strengthens the activity of NK cells and CD8+T cells, while IL‐1β recruits cytotoxic T lymphocytes (CTL) to exert antitumor effects.^9^ TNF‐ɑ, which is primarily secreted by macrophages induces HeLa cell proliferation through EMT‐mediated epithelial‐mesenchymal transformation.^10^

Current studies on the immunotherapy of cervical carcinoma have primarily focused on therapeutic vaccines targeting E6 and E7 oncoproteins^11^ and checkpoint inhibitors of PD‐1/PD‐L1.^12^ However, there are few studies on innate immunotherapy in patients with cervical carcinoma. The immunostimulant of Nocardia rubra cell‐wall skeleton (Nr‐CWS), consisting of nocardomycolic acid, arabinogalactan, and mucopeptide, enhances the ability of the host to clear infection. Nr‐CWS significantly increased the number of CD4+ and CD8+T cells in the cervical tissues of patients with HR‐HPV infection and CIN, and decreased the expression of PD‐L1.^13^ Nr‐CWS can significantly promote the activity of Cytokine‐Induced Killer cells, DCs and Natural Killer cells, which exert strong antitumor activity especially by inducing apoptosis.^14^ In addition, Nr‐CWS increases the response of CD8+T cell and activation of CD4+T cell, and promotes the differentiation of CD4+T cells into Th1 cells.^15^, ^16^ Ct26.WT tumor‐bearing mice were injected with Nr‐CWS and PD‐1 inhibitors simultaneously, which significantly inhibited the growth of transplanted tumor. This suggests that Nr‐CWS may improve the efficacy of PD‐1 inhibitors.^17^

We propose the hypothesis that Nr‐CWS influences the apoptosis of cervical carcinoma cells by promoting the antitumor effect of macrophages and dendritic cells.

## 2 MATERIALS AND METHODS

### 2.1 Patients

All studies involving human participants were reviewed and approved by the Ethics Committee of Shengjing Hospital of China Medical University (2017PS003K). The patients provided their written informed consent to participate in this study. All blood samples used in the study were from 20 cervical carcinoma patients who were treated at the Obstetrics and Gynecology Department of Shengjing Hospital of China Medical University between 2020 and 2021.

### 2.2 Mice and cell lines

Thirty female C57BL/6 mice aged 6–8 weeks were obtained from Beijing Huafukang Biosciences (China) and kept in pathogen‐free conditions. This animal study was reviewed and approved by the Ethics Committee of China Medical University (KT2020135). The human cervical carcinoma cell lines HeLa (HPV18+) (CL‐0101, Procell, China), SiHa (HPV16+) (CL‐0210, Procell, China), and the mouse cervical carcinoma cell lines U14 (CL‐0459, Procell, China), TC‐1 (CL‐0551, Procell, China) were cultured in RPMI 1640 (SH30255.01, HyClone, USA) with 10% fetal bovine serum (FBS) (164210, Procell, China). All cell lines were cultured under sterile conditions at 37°C and 5% CO_2_.

### 2.3 Cell induction and cultivation

#### 2.3.1 Dendritic cells

Using Ficoll human peripheral blood lymphocyte isolation solution (P8900, Solarbio, China), centrifuged at 800 g for 25 min to separate peripheral blood mononuclear cells (PBMC). In RPMI 1640 medium without FBS, PBMCs were cultured at 37℃ for 2 h. The non‐adherent cells were discarded. The medium was replaced with 1640 serum‐containing medium containing recombinant granulocyte‐macrophage colony‐stimulating factor (rGM‐CSF) (300–03, Peprotech, USA) (1000 U/mL) and recombinant human interleukin‐4 (IL‐4) (200–04, Peprotech, USA) (5.0 U/mL) for continuous culture for 6 days. TNF‐α (300‐01A, Peprotech, USA) (2.0 U/mL) was added on the day 7, and the culture was continued for 3 days. On the 9th day, all DCs were collected and divided into three groups, which were treated with 20 μg/mL Nr‐CWS, 30 μg/mL Nr‐CWS, and PBS for 3 days.

#### 2.3.2 Macrophage cells

The previous steps were similar to those of DCs. The medium was replaced with 1640 serum‐containing medium containing macrophage colony‐stimulating factor (M‐CSF) (315–02, Peprotech, USA) (1000 U/mL), and a half dosage was given every 2 days. On the day 7, all macrophages were collected and divided into three groups, which were treated with 20 μg/mL Nr‐CWS, 30 μg/mL Nr‐CWS, and PBS for 3 days.

#### 2.3.3 Co‐culture experiments

Transwells (3412, Corning, USA) with a 0.4‐µm aperture were used for co‐culture experiments. Mφs were cultured in the upper chamber and cervical carcinoma cells were cultured in the lower chamber. The cells were divided into four groups, untreated cervical carcinoma cells, and three other groups that were co‐cultured and treated with 20 μg/mL Nr‐CWS, 30 μg/mL Nr‐CWS, or PBS. After co‐culture for 3 days, macrophages in the upper compartment and cervical carcinoma cells in the lower compartment were collected for subsequent experiments.

### 2.4 CCK‐8

Cells in each group were collected and resuspended in 1640 medium. The cells were added to a 96‐well plate at a density of 2000 cells/well and placed at 37°C, 5% CO_2_ incubator for culture. Measuring cell viability at 0, 24, 48, 72, and 96 hours. Five parallel wells were used for different concentrations of Nr‐CWS and untreated groups. Ten microliters of CCK‐8 (HB‐CCK8‐2, Hanbio, China) reagent was added to each well and incubated at 37°C for 2 h. The absorbance was measured at 450nm with a microplate reader (1603301D, Bio Tek, USA).

### 2.5 The proportion of CD80+cells and CD68+cells by flow cytometry

We used CD80 and CD68 as markers for dendritic cells and macrophages, respectively. Cells from all groups were collected, washed twice by PBS, and suspended with 500 μL antibody buffer containing anti‐PE‐CD80 (566992, BD, USA) and FITC‐CD68(562117, BD, USA), then incubated in darkness for 20 min, and analyzed by flow cytometry (LSRFortessa, BD, The United States). Flow detection voltage settings were as follows: FSC 319, SSC 357, FITC 500, and FSC 198, SSC 242, PE 319. An antibody‐free group was set for survival control in each experiment. The gating policy is shown in Figure S1. All results were analyzed by FlowJo software.

### 2.6 Apoptosis assay

Cells from all groups were collected, washed with pre‐cooled PBS for 2 times, and resuspended with 500 µL buffer. Cell apoptosis was evaluated by apoptosis detection kit (CA1020, Solarbio, China). Annexin V‐FITC Fluorescein isothiocyanate (FITC) was added, and PI dye was added after incubation for 30 min. After 15 min of incubation, the apoptosis rate was assessed using flow cytometry. Flow detection voltage settings were as follows: FSC 181, SSC 286, FITC 341, PI 308 (DCs); FSC 181, SSC 269, FITC 286, PI 275 (Mφs); FSC 148, SSC 265, FITC 308, PI 363 (HeLa); FSC 165, SSC 269, FITC 308, PI 368 (SiHa); FSC 357, SSC 352, FITC 500, PI 478 (Hela‐si), and FSC 203, SSC 286, FITC 332, PI 407 (SiHa‐si).

### 2.7 ELISA

IL‐6 Human ELISA Kit (BMS213‐2, Invitrogen, USA), IL‐6 Mouse ELISA Kit (BMS603‐2, Invitrogen, USA), IL‐12 Human ELISA Kit (BMS2013TEN, Invitrogen, USA), IL‐12 Mouse ELISA Kit (88–7120–86, Invitrogen, USA), IL‐1β Human ELISA Kit (BMS224‐2, Invitrogen, USA), IL‐1β Mouse ELISA Kit (BMS6002, Invitrogen, USA), TNF‐ɑ Human ELISA Kit (BMS223HS, Invitrogen, USA), and TNF‐ɑ Mouse ELISA Kit (BMS607‐3, Invitrogen, USA) were used to analyze cytokines IL‐6, IL‐12, TNF‐ɑ, and IL‐1β in cell supernatant and serum according to instructions and previous studies.^18^

### 2.8 RT‐qPCR

Total RNA was extracted from cells by TRIzol reagent (16096020, Invitrogen, USA). mRNA was reverse transcribed into cDNA by the PrimeScriptTM RT Reagent Kit with gDNA Eraser (RR047A, Takara, USA). cDNA was added to the 20 µL system using SYBR Premix Ex Taq (RR820A, Takara, USA). The real‐time quantitative PCR was performed using QuantStudio (A40425, Thermo, USA). The primer sequence is shown in the Table 1. The mRNA expression was normalized to GAPDH, and relative quantification was performed by calculating 2^−ΔΔCt^. All experiments were repeated three times.

**TABLE 1:** Forward and reverse primers

Primer name	Oligo sequence (5′ to 3′)
HPV16‐E6 forward	GCAATGTTTCAGGACCCACAGGAG
HPV16‐E6 reverse	ACCTCACGTCGCAGTAACTGTTG
HPV16‐E7 forward	ATGACAGCTCAGAGGAGGAGGATG
HPV16‐E7 reverse	AACCGAAGCGTAGAGTCACACTTG
HPV18‐E6 forward	TGTGCACGGAACTGAACACT
HPV18‐E6 reverse	GCACCGCAGGCACCTTATTA
HPV18‐E7 forward	CCGACGAGCCGAACCACAAC
HPV18‐E7 reverse	CACCACGGACACACAAAGGACAG
TP53 forward	TTCCTGAAAACAACGTTCTGTC
TP53 reverse	AACCATTGTTCAATATCGTCCG
PD‐L1 forward	GCTGCACTAATTGTCTATTGGG
PD‐L1 reverse	CACAGTAATTCGCTTGTAGTCG
GAPDH forward	CGGATTTGGTCGTATTGGG
GAPDH reverse	CTGGAAGATGGTGATGGGATT

### 2.9 Colony formation assay

Cells in each group were collected and counted. Thousand cells per well were added to a 6‐well culture plate, cultured for 12 days and dyed with 0.5% crystal violet. The experiment was repeated three times. The basal planes were observed and photographed under a microscope (703548, Nikon, Japan).

### 2.10 Scratch test

Cells from each group were collected and added to a 6‐well culture plate, and cultured overnight at 37°C, 5% CO_2_ incubator. When cells had fused to 80%, the medium was discarded and the bottom of the culture plate was scratched with the tip of a 100 µL pipette. The cells were cultured in serum‐free medium for 48 h and washed with PBS. The scratch healing was observed under a microscope (703548, Nikon, Japan). The experiment was repeated three times.

### 2.11 Transwell assays

Migration and invasion experiments were carried out using Transwell chamber (3413, Corning, USA). Two hundred microliters of serum‐free RPMI 1640 containing 5*104 cells were added to the upper compartment of the Transwell chamber. The lower compartment was incubated with 800 µL 1640 medium with 10% fetal bovine serum at 37°C for 2 days. At room temperature, 4% formaldehyde was used to fix Transwell membranes for 15 min and 0.1% crystal violet was used to stain them for 25 min. Photographs were taken with an inverted microscope (703548, Nikon, Japan). The number of cells migrated to the submembrane was detected by Image‐Pro Plus 6.0. The cell invasion test was performed using the same method as the cell migration test. However, the Transwell chamber was precoated with 10 µL Matrigel (356234, BD, USA; 1/5 diluted in RPMI 1640).

### 2.12 Western Blot

Cellular and tissue proteins were harvested with RIPA lysis buffer (P0013B, Beyotime, China) and phosphatase inhibitors. BCA protein detection kit (P0012, Beyotime, China) was used to determine the protein concentration. The mixtures of 37.5 µg protein and 5X loading buffer were added to 15% SDS–polyacrylamide gels for electrophoresis. They were transferred to polyvinylidene fluoride (PVDF) (BS‐PVDF‐22, Biosharp, China) membrane after the above process. The membranes were sealed at room temperature for 1 h in 5% nonfat milk TBST buffer (20 mM Tris pH 7.4, 150 mM NaCl and 0.1% Tween‑20), and then incubated with primary antibodies at 4˚C overnight. After washing membranes with TBST buffer for 3 times, they were conjugated with the corresponding goat anti‑mouse IgG antibody (1:5000, PA1‐28555, Invitrogen, USA) or goat anti‑rabbit IgG antibody (1:5000, 31466, Invitrogen, USA) for 1 hour at room temperature. ImageJ software was used to determine the relative amount of protein using densitometry. Rabbit polyclonal anti‑GAPDH antibody (1:2500, ab9485, abcam, USA) was used as the loading control. The following primary antibodies were bounded with the proteins: mouse monoclonal anti‑HPV16+18‐E6 (1:1000, ab70, abcam, USA), rabbit polyclonal: monoclonal anti‐HPV18‐E7 (1:1000, ab100953, abcam, USA), anti‐HPV16‐E7 (1:1000, P03129, Bioss, China), anti‐p53 (1:10000, ab32389, abcam, USA), PD‐L1 (1:2000, ab205921, abcam, USA), anti‐caspase‐3 (1:5000, ab32351, abcam, USA), anti‐caspase‐8 (1:500, ab32397, abcam, USA), anti‐caspase‐9 (1:2000, ab32539, abcam, USA), anti‐BAX (1:2000, ab32503, abcam, USA), anti‐Bcl‐2 (1:1000, ab32124, abcam, USA), anti‐β‐catenin (1:1000, ab68183, abcam, USA), anti‐E‐cadherin (1:2000, ab40772, abcam, USA), anti‐N‐cadherin (1:2000, ab207608, abcam, USA), and anti‐TNFR1 (1:1000, ab259817, abcam, USA). The channels to buy antibodies were all from Shenyang Boke Biotechnology Limited Company. All experiments were repeated three times.

### 2.13 Cell cycle analysis

Cells of each group were collected and rinsed twice with PBS. The pre‐chilled ethanol was slowly added to stabilize the cells and stored for later use at −20°C. The cells were resuspended in 500 µL PI/RNase A (550825, BD, USA) staining solution and incubated in the dark for 20 min. The flow cytometry (FACSCalibur, BD, USA) was be used to analyze cell cycle. Flow detection voltage settings was as follows: FSC 319, SSC 324, and PI 429. The experiment was repeated three times.

### 2.14 Tumor growth studies

The U14 and TC‐1 tumor cells (2*106) were subcutaneously injected into C57BL/6 mice. One week later, when the average tumor volumes were 150–200 mm^3^, the mice were randomly assigned. All mice were randomly divided into three groups (5 mice/each group): (A) the control group, which received PBS (100 μL); (B) the Nr‐CWS‐low group, which received 5 μg Nr‐CWS (100 μL); (C). the Nr‐CWS‐high group, which received 10 μg Nr‐CWS (100 μL). Tumor size was measured with Vernier caliper every 3 days, and formula to calculate tumor volume as follows: Volume = 0.5* (width)^2^ * (length).

### 2.15 Immunohistochemistry and image analysis

Fresh tumor tissues were stabilized in 4% paraformaldehyde and embedded in paraffin blocks. The immunohistochemistry kit (E‐IR‐R211, Elabscience, China) was used to analyze tissue sections (4 μm thick). Slides were incubated overnight at 4°C with following primary antibodies: mouse anti‐human: HPV16+18‐E6 (1:100, ab70, abcam, USA), HPV18‐E7 (1:100, ab100953, abcam, USA), rabbit anti‐human: HPV16‐E7 (1:100, P03129, Bioss, China), P16 (1:100, ab108349, abcam, USA), Ki67 (1:100, ab15580, abcam, USA), and PCNA (1:100, ab18197, abcam, USA). The microscope (703548, Nikon, Japan) was used to observe and photograph the slides.

### 2.16 Immunofluorescence

Paraffin sections of 6 μm thick tissue were fixed and blocked in 10% BSA for 2 h. Sections were incubated overnight with mouse anti‐human: HPV16+18‐E6 (1:100, ab70, abcam, USA), HPV18‐E7 (1:100, ab100953, abcam, USA), and rabbit anti‐human: HPV16‐E7 (1:100, P03129, Bioss, China) at 4℃. Immunofluorescence staining was performed using Goat polyclonal Secondary Antibody to Rabbit/Mouse IgG‐H&L (1:200, ab150079/ ab150115, abcam, USA) and 4’, 6‐diamino‐2‐phenylindoles (DAPI) (S2110, Solarbio, China). The sections were observed by confocal microscopy. The microscope (703548, Nikon, Japan) was used to observe and photograph the slides.

### 2.17 Transfection

Small‐interfering RNAs (siRNAs) against TNFR1 and control siRNA were purchased from Hanbio (China). siTNFR1 and siRNA transfections were carried out using Lipofectamine 2000 (11668019, Invitrogen, USA) according to the instructions.

### 2.18 Statistical analysis

Calculations were performed using GraphPad Prism 8.0 version. The mean ± SD of at least three independent trials represented all data. Student's *t*‐test analysis was used for comparison between the two groups, and one‐way ANOVA with the least significant difference (LSD) test was used for the comparison of multiple groups. A *p* < 0.05 was considered a statistically significant difference.

## 3 RESULTS

### 3.1 Cell identification and optimum conditions

**FIGURE 1:**
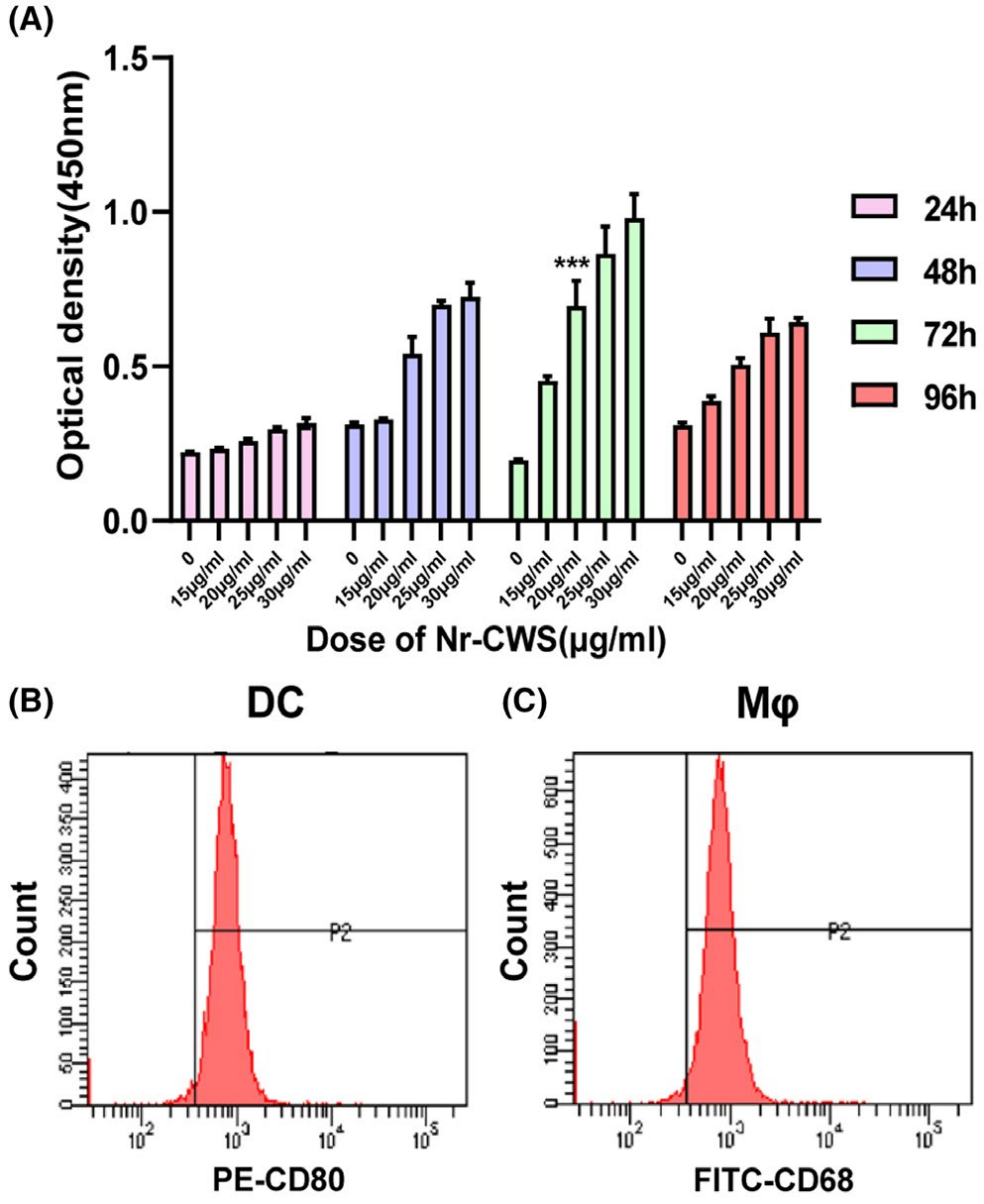
The induction and identification of DCs and Mφs and the determination of the optimal concentration and optimal time of Nr‐CWS. (A) The percentage of CD80+DCs determined by FCM. (B) The percentage of CD68+Mφs determined by FCM. (C) CCK8 was used to detect that the optimal concentration of Nr‐CWS on PBMC was 20μg/ mL and the optimal time was 72h. (*p < 0.05, **p < 0.01, ***p < 0.001)

CCK‐8 was used to determine the optimal concentration and optimal action time of Nr‐CWS on immune cells. We found that Nr‐CWS increased PBMC activity in a dose‐dependent and time‐dependent manner. Under the optimal concentration of 20 μg/mL, 72 h was the optimal administration condition of Nr‐CWS (Figure 1A). CD80 molecules are highly expressed in mature dendritic cells.^19^ CD68 molecules are recognized as universal markers of macrophages.^20^ After inducing factor stimulation, the proportions of dendritic cells and macrophages were 97.3% and 96.9%, respectively (Figure 1B,C).

### 3.2 Nr‐CWS enhances vitality and decreases the expression of PD‐L1 in DCs and Mφs

**FIGURE 2:**
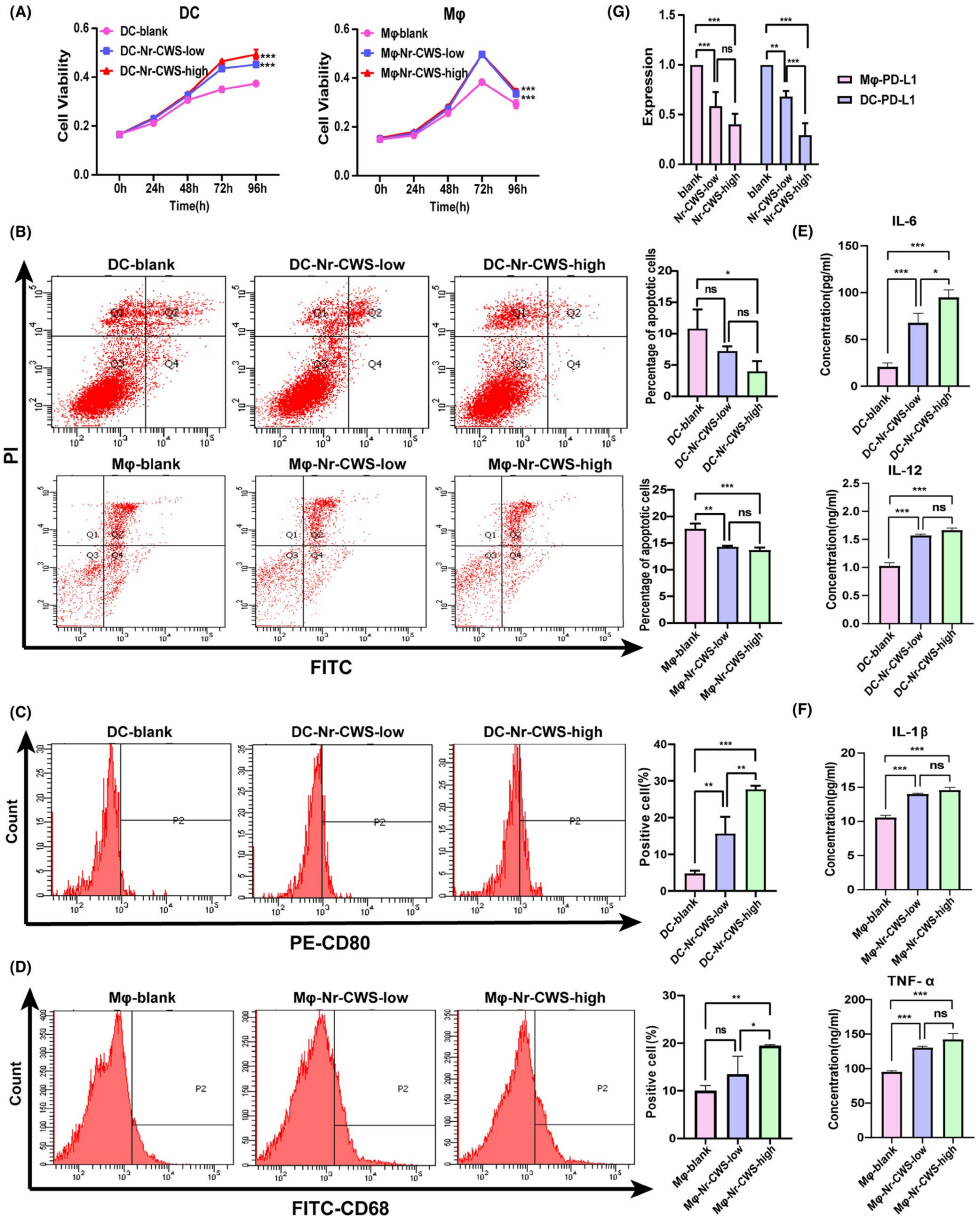
Effects of Nr‐CWS on DCs and Mφs. (A) CCK8 was used to detect that the proliferation of DCs and Mφs was induced by Nr‐CWS. (B) FCM was used to detect that Nr‐CWS could significantly reduce the apoptosis of DCs and Mφs. (C) FCM was used to detect that Nr‐CWS could promote the induction of CD80+DCs by PBMC. (D) FCM was used to detect that Nr‐CWS could promote the induction of CD68+Mφs by PBMC. (E) ELISA was used to detect that Nr‐CWS could increase the content of cytokines IL‐6 and IL‐12 secreted by DCs. (F) ELISA was used to detect that Nr‐CWS could increase the content of cytokines IL‐1β and TNF‐ɑ secreted by Mφs. (G) RT‐qPCR was used to detect that Nr‐CWS could increase the expression of PD‐L1 in DCs and Mφs. (*p < 0.05, **p < 0.01, ***p < 0.001)

Three groups of DCs and Mφs were cultured using two concentrations of Nr‐CWS and PBS. After culture, the proliferation and apoptosis rates of DCs and Mφs treated with Nr‐CWS were measured. Compared with non‐Nr‐CWS group, different concentrations of Nr‐CWS changed DCs and Mφs in terms of promoting proliferation and reducing the rate of apoptosis (Figure 2A,B). The high concentration of Nr‐CWS significantly reduced the apoptosis rate of dendritic cells and macrophages by 63% and 22%, respectively. To determine whether Nr‐CWS increases the expression of CD80+DCs and CD68+Mφs, flow cytometry was used to detect the expression of CD80+DCs and CD68+Mφs in PBMCs after Nr‐CWS treatment. The results showed that high concentration of Nr‐CWS could increase the number of CD80+DCs and CD68+Mφs in PBMC by 82.7% and 48.5%, respectively (Figure 2C,D). To further explore the effects of Nr‐CWS on DCs and Mφs, cytokines in the supernatants of induced macrophages and dendritic cells were detected. The results indicated that Nr‐CWS simultaneously increased secretion of the cytokines IL‐6 and IL‐12 by DCs and the cytokines TNF‐ɑ and IL‐1β by Mφs (Figure 2E,F). Further results showed that in the three groups of DCs and Mφs, PD‐L1 expression levels were strikingly reduced in response to administration of Nr‐CWS. Especially, high concentration of Nr‐CWS can reduce the expression of PD‐L1 RNA in macrophages and dendritic cells by 60% and 71% (Figure 2G).

### 3.3 The biological function of co‐cultured cervical carcinoma cells changes after the addition of Nr‐CWS

**FIGURE 3:**
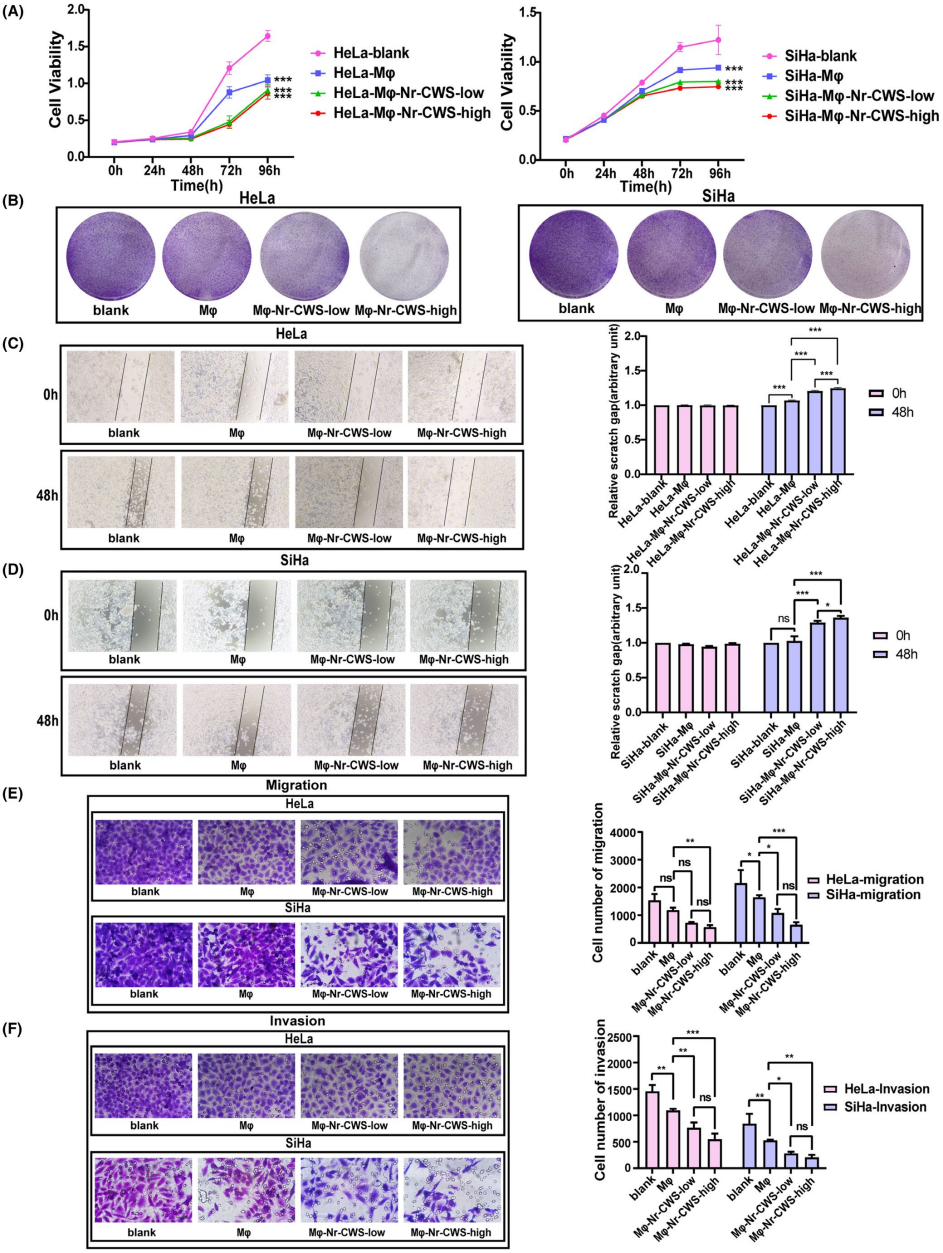
Effect of Nr‐CWS on biological function of co‐cultured cervical carcinoma cell lines. (A) Using CCK8 to detect Nr‐CWS can significantly reduce the proliferation of co‐cultured HeLa and SiHa cell lines. (B) Nr‐CWS could reduce the colony‐forming ability of co‐cultured HeLa and SiHa cell lines. (C, D) Scratch test was used to detect that Nr‐CWS could reduce the migration ability of co‐cultured HeLa and SiHa cell lines. (E, F) Transwell test was used to detect that Nr‐CWS could reduce the migration and invasion ability of co‐cultured HeLa and SiHa cell lines. (*p < 0.05, **p < 0.01, ***p < 0.001)

It was found that the proliferation rate of co‐cultured cervical carcinoma cells cultured with Nr‐CWS was found to be significantly lower than that of the corresponding control media through CCK‐8 experiment (Figure 3A) and colony formation experiment (Figure 3B). Scratch assays (Figure 3C,D) and Transwell assays (Figure 3E,F) identified Nr‐CWS‐mediated decreases in the migration and invasion abilities of co‐cultured cervical carcinoma cells.

### 3.4 Nr‐CWS affects the expression of E6 and E7 oncogenes and related genes in co‐cultured cervical carcinoma cells, affecting their cell cycle

**FIGURE 4:**
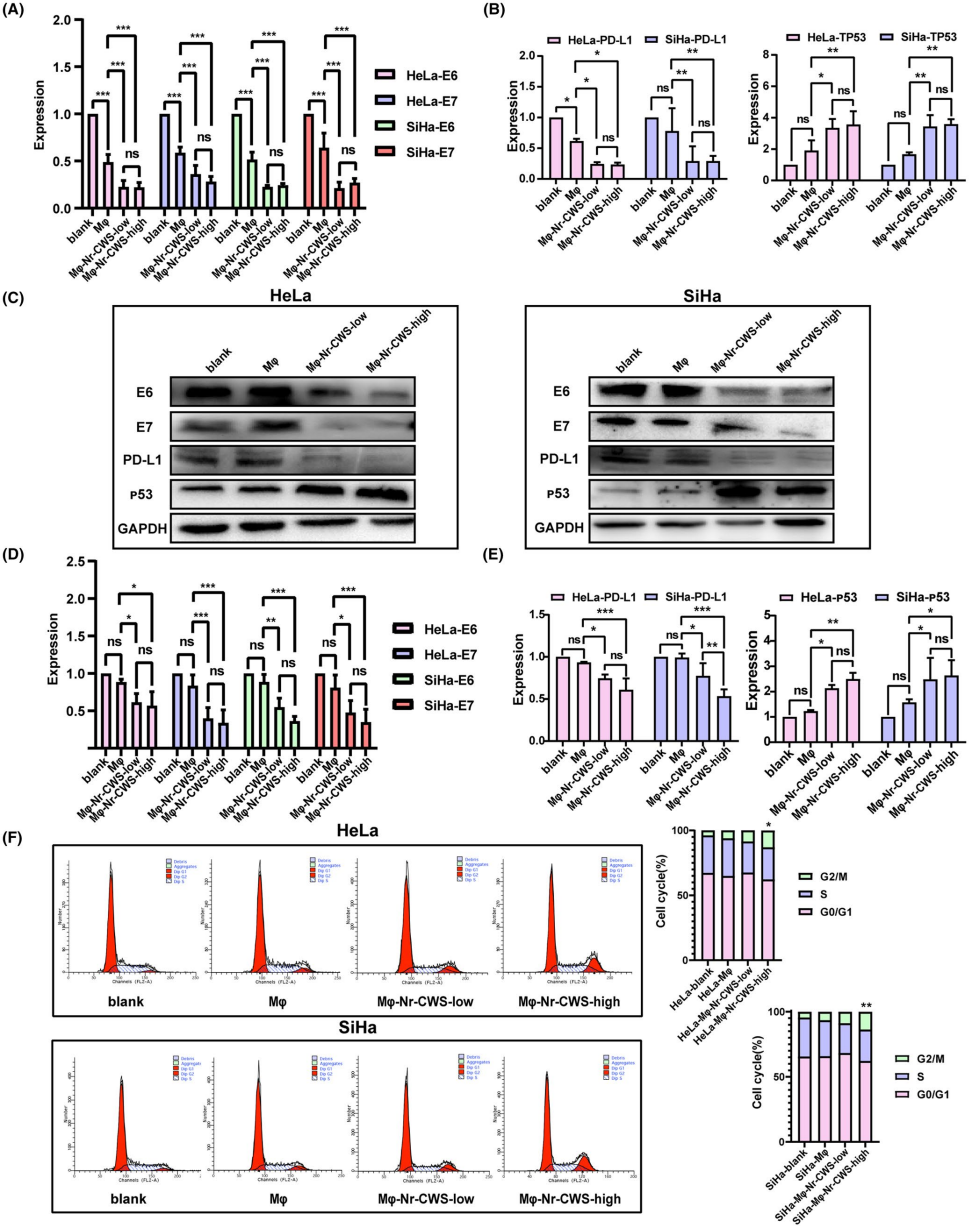
Effects of Nr‐CWS on cell cycle and related genes of co‐cultured cervical carcinoma cell lines. (A, B) RT‐qPCR was used to detect that Nr‐CWS could reduce the mRNA expression of E6, E7 and PD‐L1, and increase the mRNA expression of TP53 in co‐cultured cervical carcinoma cell lines. (C–E) Western Blot detected that Nr‐CWS could reduce the protein expression of E6, E7 and PD‐L1, and increase the protein expression of TP53 in co‐cultured cervical carcinoma cell lines. (F) FCM detected that more HeLa and SiHa cells entered G2/M phase, after the addition of Nr‐CWS. (*p < 0.05, **p < 0.01, ***p < 0.001)

We next investigated whether Nr‐CWS affects the expression of cervical carcinoma oncogenes E6 and E7 by enhancing the antitumor effect of macrophages. RT‐qPCR and Western Blot were used to determine the expression of E6 and E7 in the HeLa/SiHa‐blank groups, HeLa/SiHa‐Mφ groups, HeLa/SiHa‐Mφ‐Nr‐CWS‐low groups, and HeLa/SiHa‐Mφ‐Nr‐CWS‐high groups. The results revealed that expression of E6 and E7 in HeLa/SiHa‐Mφ‐Nr‐CWS‐low groups and HeLa/SiHa‐Mφ‐Nr‐CWS‐high group was significantly decreased compared to HeLa/SiHa‐Mφ groups. Among them, the expression of E6 RNA in HeLa/SiHa‐Mφ‐Nr‐CWS‐low groups decreased by 54% and 56%, and the expression of E7 RNA in HeLa/SiHa‐Mφ‐Nr‐CWS‐high groups decreased by 52% and 53%. However, the expression of E6 and E7 genes in cervical carcinoma cells was not related to the dose of Nr‐CWS (Figure 4A,C,D). Obviously, we detected the expression of tumor suppressor gene TP53 and the immune checkpoint gene PD‐L1 in the four groups of cervical carcinoma cells. The experiments demonstrated that the levels of TP53 were increased 1.87 and 2.15 times and levels of PD‐L1 were decreased 62% and 63% in response to Nr‐CWS consistent with the expression results of E6 and E7 (Figure 4B,C,E).

To investigate whether Nr‐CWS can affect the cycle of cervical carcinoma cells, Nr‐CWS or PBS was used to treat co‐cultured HeLa and SiHa cells. We found that Nr‐CWS significantly increased the proportion of G2/M phase cells (Figure 4F).

### 3.5 Nr‐CWS promotes the apoptosis of co‐cultured HeLa and SiHa cells

**FIGURE 5:**
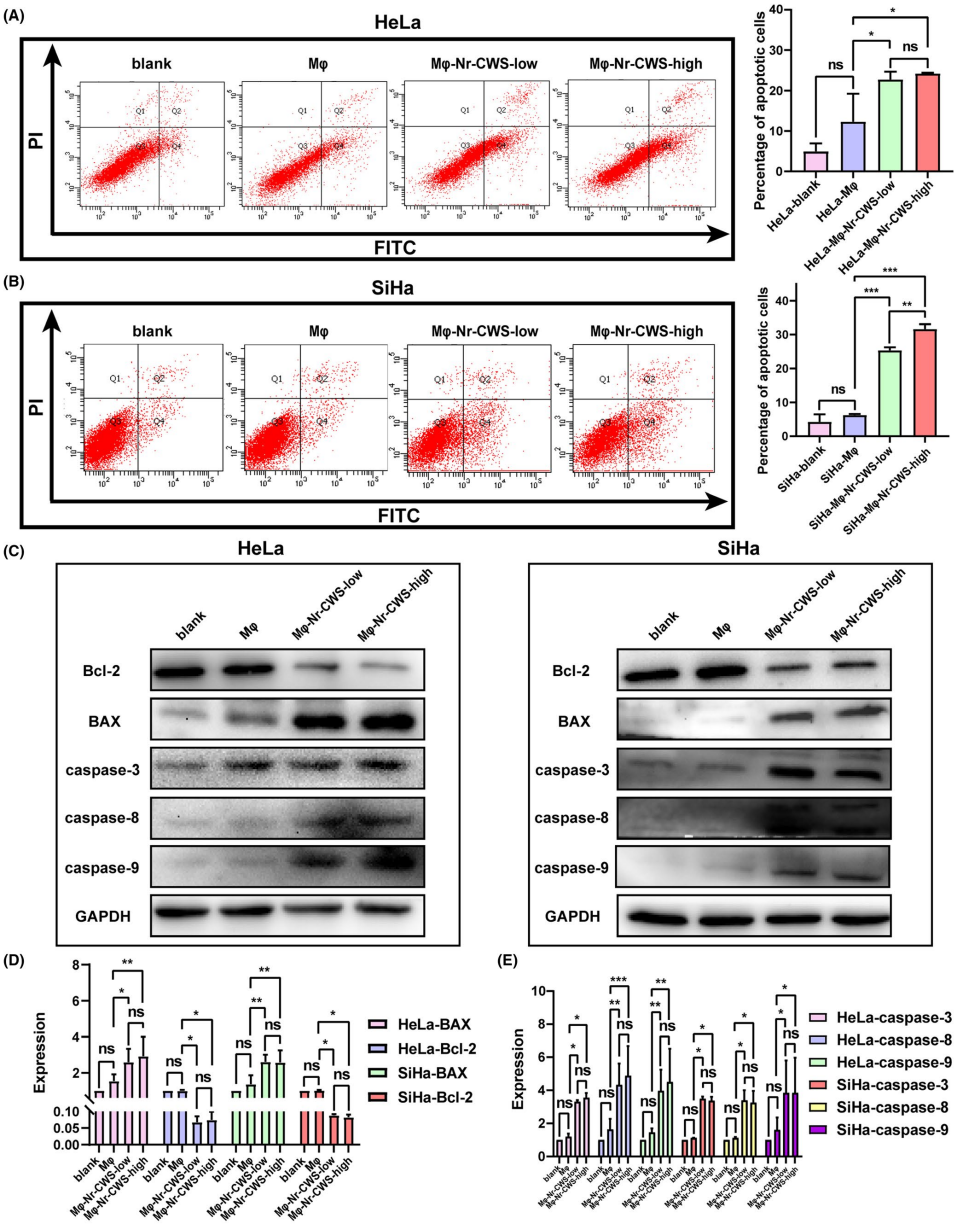
Effect of Nr‐CWS on apoptosis of co‐cultured cervical carcinoma cell lines. (A, B) FCM detected that after adding Nr‐CWS, the apoptosis rate of co‐cultured HeLa and SiHa cells increased. (C–E) Western Blot detected the increase in BAX, caspase‐3, caspase‐8 and caspase‐9 protein expression levels in HeLa and SiHa co‐cultured with Nr‐CWS, while the Bcl‐2 protein expression level decreased. (*p < 0.05, **p < 0.01, ***p < 0.001)

The apoptosis of co‐cultured HeLa and SiHa cells treated with Nr‐CWS was detected by flow cytometry. We found that Nr‐CWS increased the apoptosis rate of co‐cultured HeLa and SiHa cells. Compared with the HeLa/SiHa‐Mφ groups, the apoptosis rate of HeLa/SiHa‐Mφ‐Nr‐CWS‐low groups were increased 1.84 and 4.06 times, and the apoptosis rate of HeLa/SiHa‐Mφ‐Nr‐CWS‐high groups were increased 1.96 and 5.08 times. (Figure 5A,B). The levels of Bcl‐2, BAX, caspase‐3, caspase‐8, and caspase‐9 protein were detected by western blot. Compared to the PBS control group, the levels of Bcl‐2 protein in co‐cultured HeLa and SiHa cells treated with Nr‐CWS were significantly decreased, while the levels of BAX, caspase‐3, caspase‐8, and caspase‐9 protein were significantly increased (Figure 5C,D,E). These results indicate that Nr‐CWS promotes the apoptosis in co‐cultured cervical carcinoma cells.

### 3.6 Nr‐CWS inhibits the activation of the Wnt/β‐catenin and EMT pathways

**FIGURE 6:**
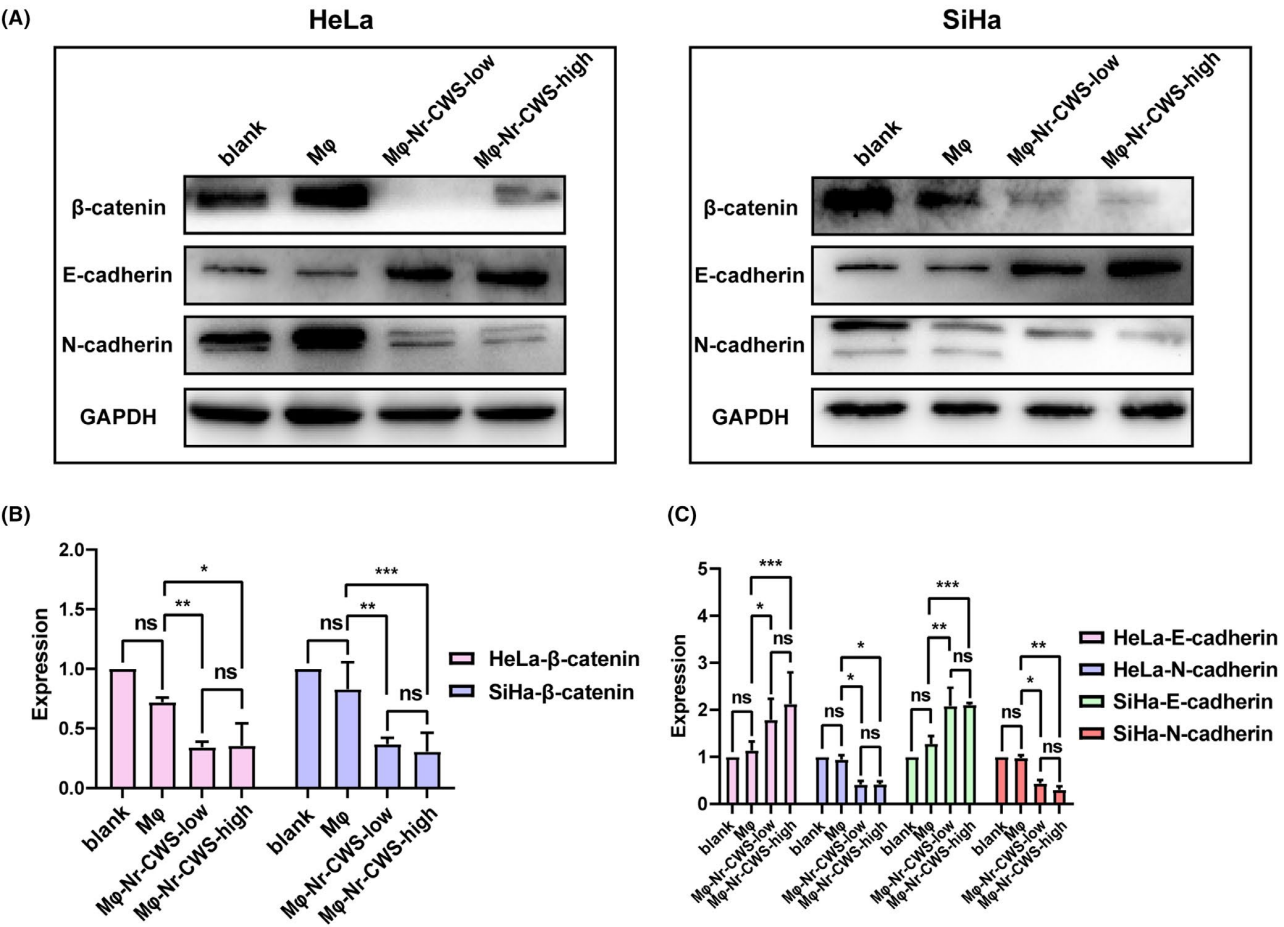
Effect of Nr‐CWS on Wnt/β‐catenin‐EMT pathway in co‐cultured cervical carcinoma cell lines. (A,B) Western Blot detected the decrease of β‐catenin protein expression levels in HeLa and SiHa co‐cultured with Nr‐CWS. (A, C) Western Blot detected the increase in E‐cadherin protein expression levels in HeLa and SiHa co‐cultured with Nr‐CWS, while the N‐cadherin protein expression level decreased. (*p < 0.05, **p < 0.01, ***p < 0.01)

It has been reported that β‐catenin, E‐cadherin, and N‐cadherin are key factors regulating tumor development.^21^ Western blot was also used to detect the effects of Nr‐CWS on Wnt/β‐catenin and EMT pathways. It was found that in the absence of Nr‐CWS, β‐catenin, N‐cadherin, and E‐cadherin were not significantly different in HeLa and SiHa cells co‐cultured with M compared with the blank group. However, after the addition of different doses of Nr‐CWS, the content of β‐catenin, N‐cadherin in cells decreased significantly, while the content of E‐cadherin increased significantly. However, this trend seems to be independent of the dose of Nr‐CWS (Figure 6A–C).

### 3.7 In vivo experiments demonstrate that Nr‐CWS promotes the antitumor immune response of DCs and Mφs

**FIGURE 7:**
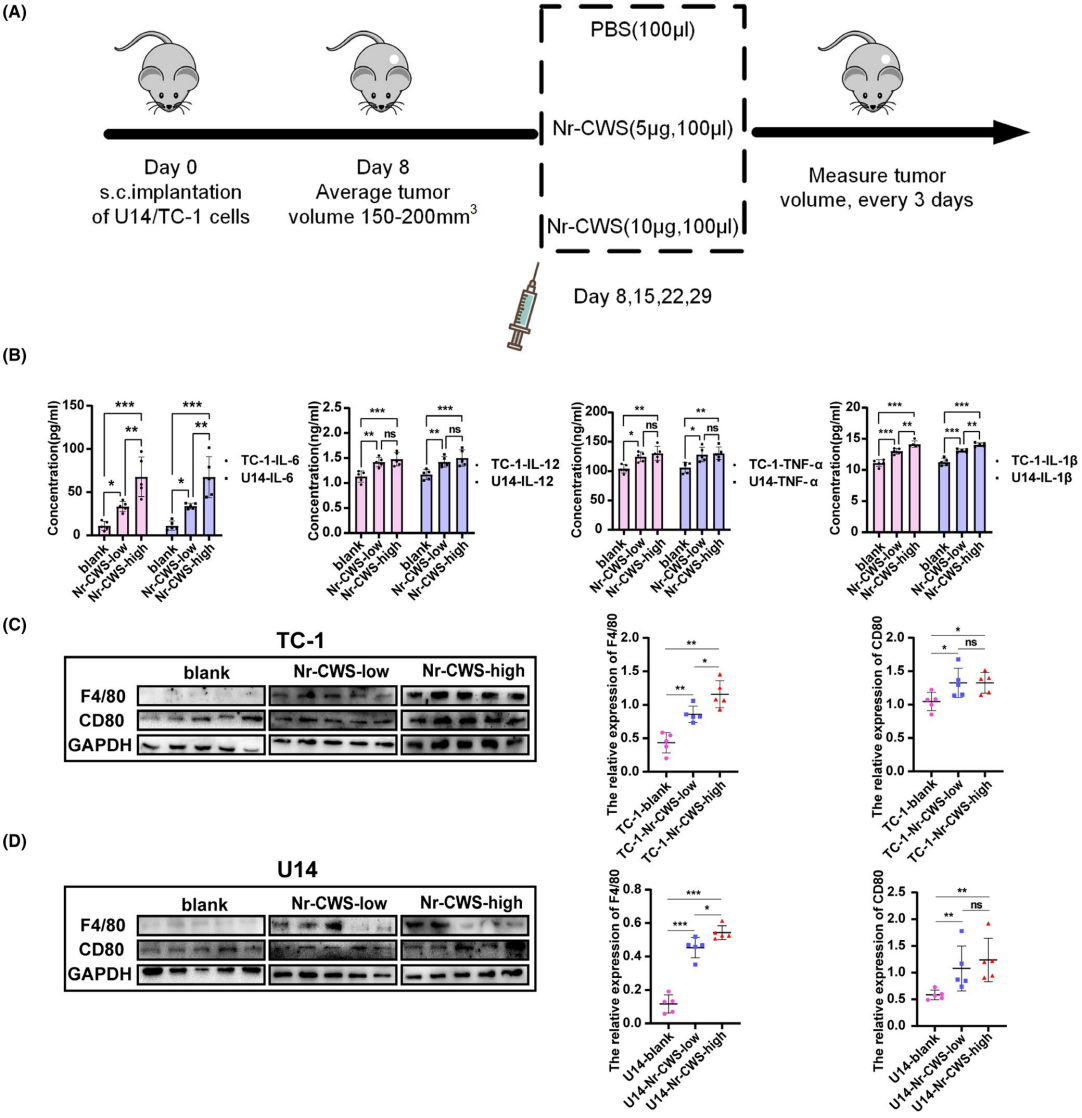
Effect of Nr‐CWS on the activity of DCs and Mφs in tumor‐bearing mice. (A) Schedule of in vivo experiments. (B) After the tumor‐bearing mice were treated with Nr‐CWS, the serum levels of IL‐6, IL‐12, TNF‐ɑ and IL‐1β increased by ELISA. (C) Western Blot detected that Nr‐CWS increased the expression of F4/80 and CD80 proteins in mice subcutaneous tumors induced by TC‐1. (D) Western Blot detected that Nr‐CWS increased the expression of F4/80 and CD80 proteins in mice subcutaneous tumors induced by U14. (*p < 0.05, **p < 0.01, ***p < 0.001)

To investigate whether Nr‐CWS enhances the antitumor effect of DCs and Mφs in vivo, mice were randomly divided into groups, and started treatment when the average tumor volume reached 150–200 mm^3^ (*n* = 5). Mice were intraperitoneally injected with either Nr‐CWS or PBS on days 8, 15, 22, and 29 (Figure 7A). On day 36, the levels of cytokines in mouse venous blood were measured. Similar to the results of the in vivo experiments, compared with the PBS group, IL‐6, IL‐12, TNF‐ɑ, and IL‐1β levels in the blood of mice in the Nr‐CWS group increased significantly. Of note, IL‐6 and IL‐1β levels increased in a dose‐dependent manner in vivo. Compared with the control group, IL‐6 levels in the Nr‐CWS‐low groups were increased by 3 times, and that in the Nr‐CWS‐high groups were increased by 6 times, while IL‐1β levels were approximately 17.2% and 24.6% higher (Figure 7B). Next, expression levels of F4/80, CD80, and TNF‐ɑ in the tumor tissues of mice in each group were detected by western blot. The results showed that the levels of F4/80, CD80, and TNF‐ɑ protein in tumor tissues of the Nr‐CWS treatment group were higher than those in the PBS group (Figure 7C,D).

### 3.8 Effect of Nr‐CWS on subcutaneous tumor in mice

**FIGURE 8:**
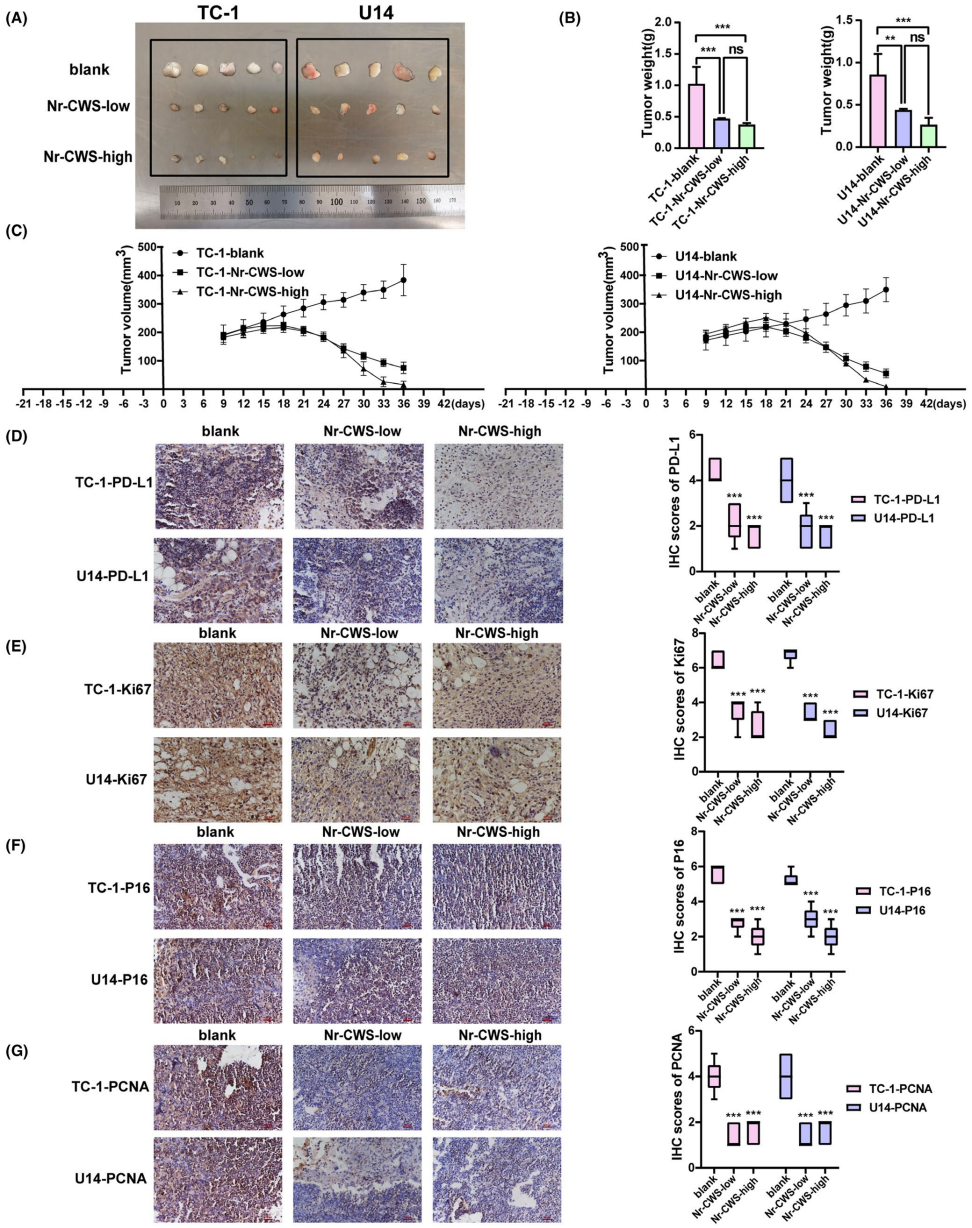
Effect of Nr‐CWS on subcutaneous tumor growth in mice. (A) Tumor image of each group of tumor‐bearing mice. (B, C) Nr‐CWS could significantly inhibit the mass and volume of subcutaneous tumor growth in mice. (D) Immunohistochemistry was used to detect that Nr‐CWS could reduce the expression of PD‐L1 in mice tumors. (E) Immunohistochemistry was used to detect that Nr‐CWS could reduce the expression of Ki67 in mice tumors. (F) Immunohistochemistry was used to detect that Nr‐CWS could reduce the expression of P16 in mice tumors. (G) Immunohistochemistry was used to detect that Nr‐CWS could reduce the expression of PCNA in mice tumors. (*p < 0.05, **p < 0.01, ***p < 0.001)

**FIGURE 9:**
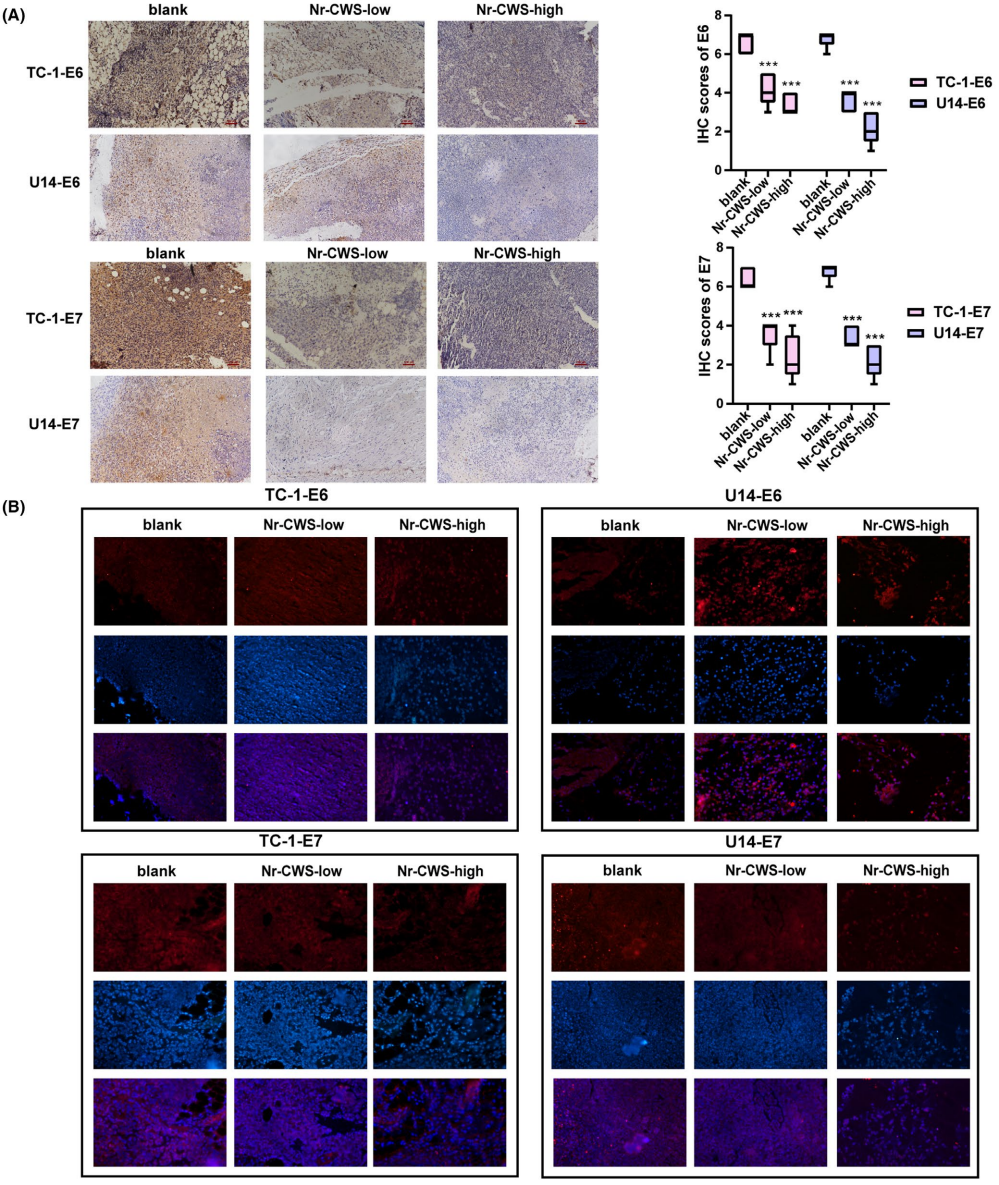
Effect of Nr‐CWS on the expression of E6 and E7 oncoproteins in tumor tissues of mice. (A) Immunohistochemistry was used to detect that Nr‐CWS could significantly reduce the expression of E6 and E7 oncoproteins in mouse tumor tissues. (B) Immunofluorescence was used to detect that Nr‐CWS could significantly reduce the expression of E6 and E7 oncoproteins in mouse tumor tissues

As shown in Figure 8, the tumor weight and volume in the control group were higher than those in the Nr‐CWS treatment group. Compared with TC‐1/U14‐blank groups, tumors volume decreased by 53% and 49% in the TC‐1/U14‐Nr‐CWS‐low groups, and 62% and 69% in the TC‐1/U14‐Nr‐CWS‐high groups (Figure 8A–C). These results showed that Nr‐CWS inhibited the growth of cervical carcinoma tumors in mice. Subsequently, we detected the expression of E6 and E7 in mouse tumor tissues by immunohistochemistry and immunofluorescence. Expression of E6 and E7 oncoproteins in tumor tissues of mice in the Nr‐CWS treatment group was significantly decreased (Figure 9A,B). At the same time, compared to the control group, PD‐L1 and some proliferation indicators also exhibited downward trend (Figure 8D–G).

### 3.9 After knocking down TNFR1, the ability of Nr‐CWS to promote apoptosis of cervical carcinoma cells decreased

**FIGURE 10:**
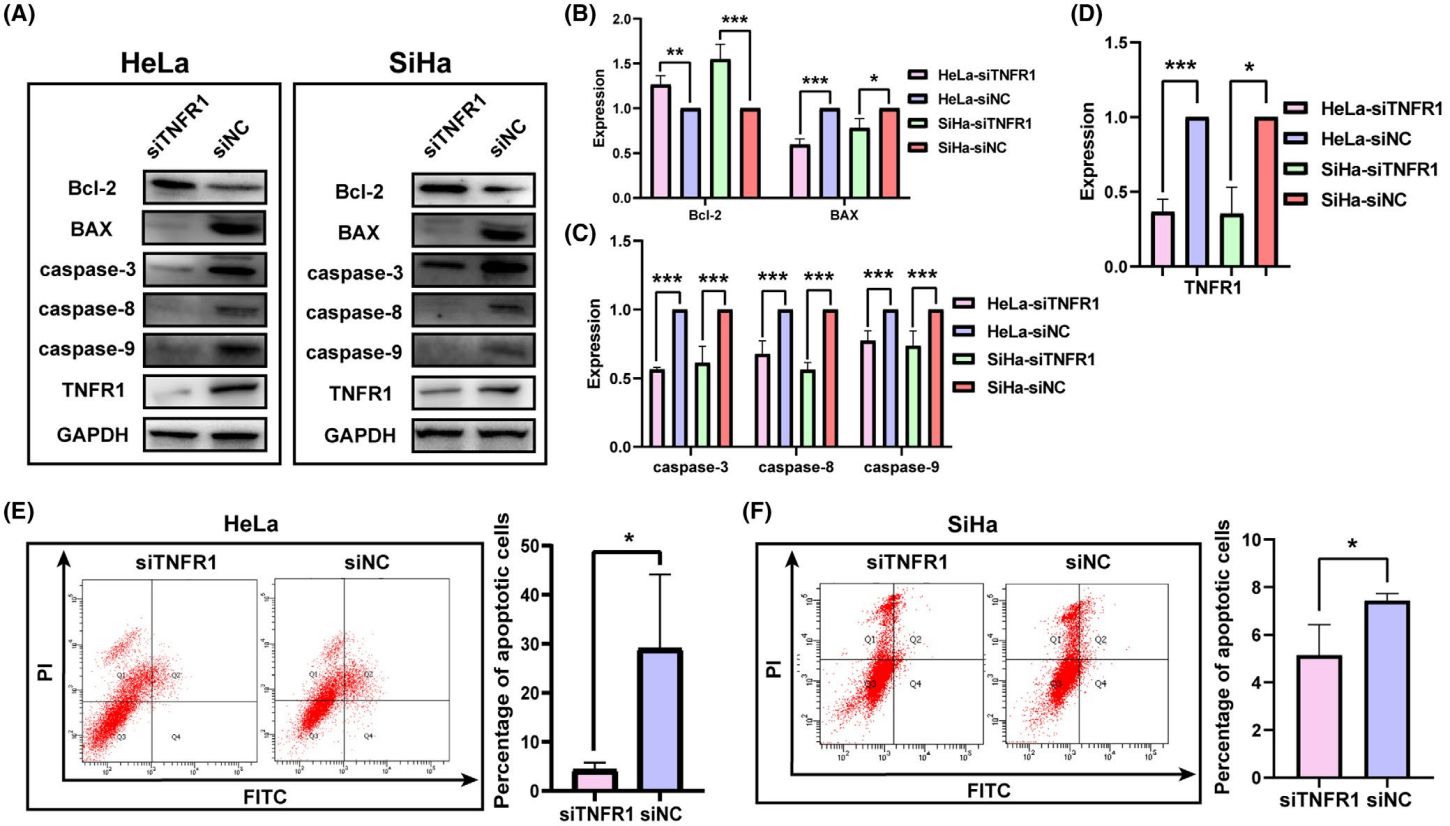
After knocking down TNFR1, the ability of Nr‐CWS to promote apoptosis of cervical carcinoma cells decreased. (A–D) Western Blot detected that the expression levels of BAX, caspase‐3, caspase‐8, caspase‐9 and TNFR1 proteins in co‐cultured HeLa and SiHa transfected with siTNFR1 and siNC. (E, F) FCM detected that the apoptosis rate of co‐cultured HeLa and SiHa transfected with siTNFR1 and siNC

In order to verify whether Nr‐CWS promotes apoptosis of co‐cultured cervical carcinoma cells because of increased secretion of TNF‐ɑ by macrophages. Cervical carcinoma cell lines transfected with siTNFR1 and siNC were co‐cultured with Mφs in the presence of Nr‐CWS. Compared with siNC groups, the level of Bcl‐2 protein increased in siTNFR1 groups while expressions of BAX, caspase‐3, caspase‐8, and caspase‐9 protein decreased (Figure 10A–D). At the same time, compared to the siNC groups, the apoptosis rate of siTNFR1 groups was reduced by 86.3% and 31.0% by flow cytometry, respectively (Figure 10E,F).

**FIGURE 11:**
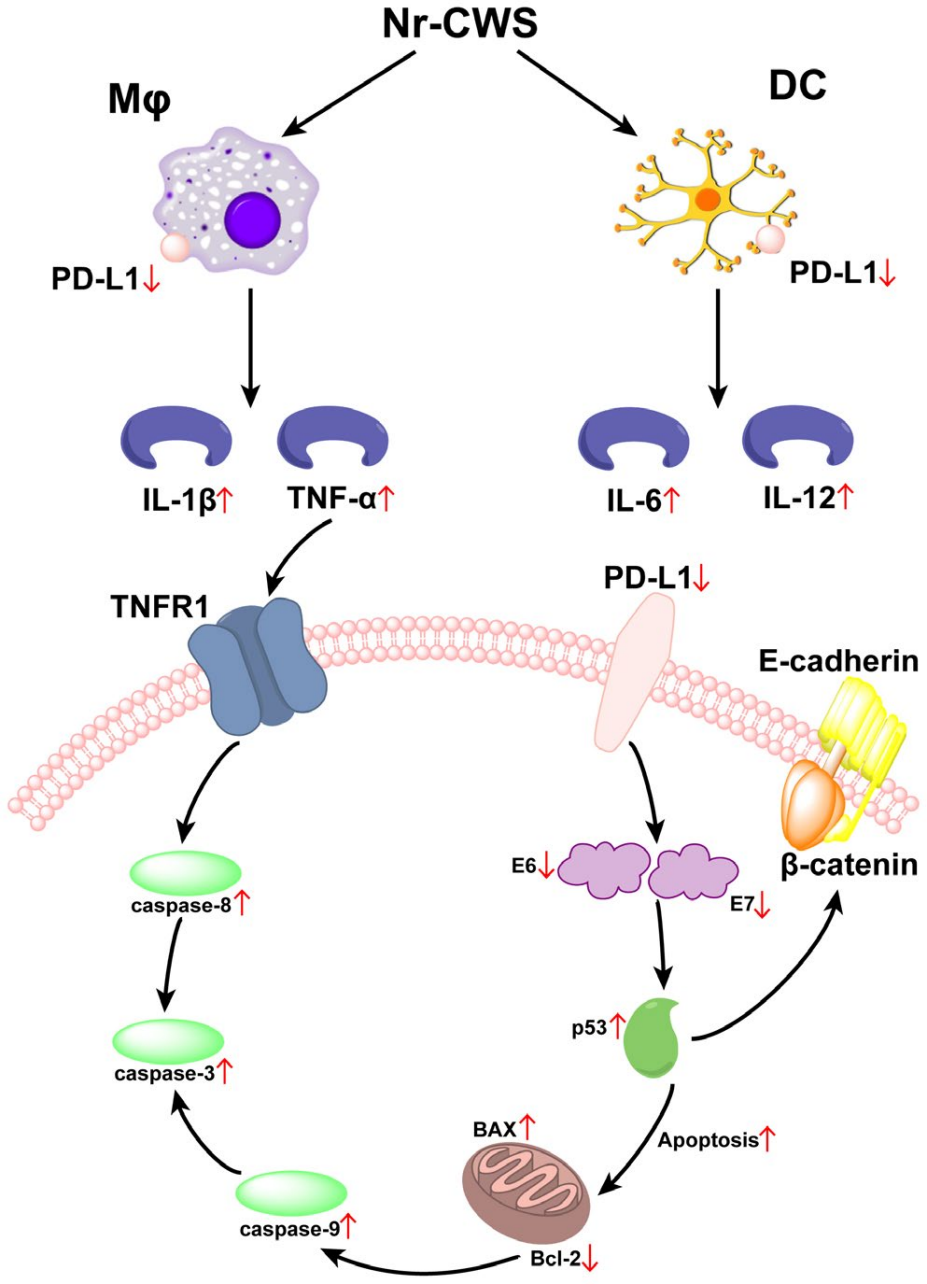
Schematic illustration represented the inhibitory effect of Nr‐CWS on cervical carcinoma

All the results confirm that, Nr‐CWS can promote apoptosis of cervical carcinoma cells by enhancing the antitumor effect of dendritic cells and macrophages (Figure 11).

## 4 DISCUSSION

Immunotherapeutic approaches for cervical carcinoma are primarily divided into four aspects: immunomodulators, inhibitors of immune checkpoints and action sites, CAR‐T cell therapy, and therapeutic vaccines against E6 and E7.^22^ Among them, immunomodulators, such as Inosine Pranobex (IP) immunotherapy,^23^, ^24^ are current topic of interest. Nr‐CWS is not only a biological response regulator, but also an immunomodulator. Whether Nr‐CWS can be used to treat patients with cervical carcinoma remains an open question.

Tumor‐associated macrophages (TAMs) are the most important cells in the tumor microenvironment (TME) of cervical carcinoma.^25^ Studies have shown that the density of CD68+TAM in cervical carcinoma tissue is higher than that in adjacent tissue and normal tissue. A high density of CD68+TAMs is associated with lymph node metastasis.^26^ Under normal circumstances, dendritic cells promote the antitumor immune response of Th1/CTL and enhance the immune monitoring of tumor cells by secreting IL‐12. Studies have confirmed that an HPV16E5 protein‐targeted dendrite vaccine used in the treatment of tumor mice, controlled the growth of mouse tumors and improved the survival rate of mice.^27^ We first investigated whether Nr‐CWS enhanced the activity and antitumor function of dendritic cells and macrophages. We demonstrated that Nr‐CWS increases the activity of dendritic cells and macrophages and inhibit their apoptosis.

Next, to investigate whether Nr‐CWS affects the secretion function of dendritic cells and macrophages, the IL‐6, IL‐12, TNF‐ɑ, and IL‐1β levels in culture medium and mouse serum were determined. The secretion of these four cytokines was increased by the addition of both low and high doses of Nr‐CWS. Among them, the increasing trends of IL‐12 and TNF‐ɑ were consistent with the study results of Wei et al. in high‐risk HPV infection and cervical intraepithelial neoplasia.^13^ The trends in IL‐6 and IL‐1β levels in patients with bladder cancer were similar to the results of the study by Elizabeth et al. However, none of their studies indicated the origin of the increased cytokines.^28^ Our experiments suggest that these increased cytokines may come from the increase in the activity of dendritic cells and macrophages. The role of IL‐6 in disease is bidirectional. On the one hand, activation of the IL‐6‐STAT3 signaling pathway promotes the epithelial‐mesenchymal transformation and metastasis of tumors; on the other hand, IL‐6 and IL‐1β are the driving forces of Th17 polarization, which can enhance the role of resistance against extracellular pathogens.^29^ Since cervical carcinoma patients often have persistent HPV infection, Nr‐CWS may promote the function of IL‐6 and IL‐1β against foreign pathogens. In adoptive cell therapy, pretreatment of CD8+T by IL‐12 enhances the function of tumor‐reactive CD8+T cells.^30^ TNF‐ɑ can bind to tumor necrosis factor receptor 1 (TNFR1) and initiate apoptosis through activation of the caspase cascade under certain conditions.^31^

After addition of Nr‐CWS, the expression of PD‐L1 in dendritic cells, macrophages, cervical carcinoma cell lines, and mouse tumor tissues was significantly decreased. Previous studies have proven that the expression of PD‐L1 in cervical carcinoma tissues is higher than that in normal cervical tissues.^1^ After the expression of PD‐L1 in tissues and PD‐1 protein on T cells is induced by cytokines, immune tolerance can be induced. When this immune tolerance is activated, T cell receptor (TCR) signaling is inhibited, thereby inhibiting T cell activity.^32^ Studies by TAO et al. and Wang et al. confirmed that Nr‐CWS promotes the proliferation of CD4+T cells and increase the response of CD8+T cells. The possible mechanism involves Nr‐CWS reducing the expression levels of PD‐L1 and attenuating the immunosuppression. Based on these results, we hypothesize that Nr‐CWS enhances the antitumor effect of dendritic cells and macrophages.^15^, ^16^

Next, we investigated whether this enhanced antitumor effect inhibits the development of cervical carcinoma. According to the study of Meng et al., we co‐cultured macrophages induced by PBMCs with cervical carcinoma cell lines HeLa and SiHa.^14^ We found that the abilities of cervical carcinoma cells to proliferate, invade, and migrate were reduced in response to Nr‐CWS. This shows that Nr‐CWS affects the biological function of cervical carcinoma cell lines by increasing the antitumor effect of macrophages. Wnt signaling, especially Wnt/β‐catenin signaling, plays a switching role in the occurrence, metastasis, recurrence, and chemotherapy resistance of cervical carcinoma.^33^ β‐catenin is encoded by CTNNB1 and is a multifunctional protein that regulates the cell growth and intracellular adhesion in epithelial cells. Cadherin is transmembrane glycoprotein responsible for the adhesion between cells and the maintenance of normal tissue structure. Among them, E‐cadherin and N‐cadherin are two classic cadherins.^34^ Cadherin usually binds to β‐catenin at the tail end of the cytoplasm and aggregates into a connective structure called the cadherin‐catenin complex. Dysfunction of this complex occurs in tumors, resulting in an increase in β‐catenin in the cytoplasm and nucleus. Catenin mediates many signals transduction processes. They regulate tumor growth, metastasis, and epithelial‐mesenchymal transformation.^21^ We detected the changes in β‐catenin, E‐cadherin, and N‐cadherin protein expression in cervical carcinoma cell lines after adding Nr‐CWS. The decrease in β‐catenin, and N‐cadherin and the increase in E‐cadherin indicate the "reversal" of tumor development in response to Nr‐CWS administration.

During the process of HPV infection, viral DNA is integrated into the host genome, and viral oncoproteins E6 and E7 are expressed in cervical epithelial cells, interacting with cellular proteins.^35^ The well‐known carcinogenic mechanism involves E6 and E7 combining and inactivating the p53 and retinoblastoma protein (pRb), respectively, blocking the normal regulation of the cell cycle, and initiating the transcription of oncogenes.^36^ E6 and E7 interact with proteins in many signal transduction pathways that are involved in the formation of malignant phenotypes, including β‐catenin. Lichtig et al. confirmed for the first time that HPV16‐E6 enhanced β‐catenin/Tcf transcription in the form of E6‐related protein (E6AP), providing a possible mechanistic link between the E6 oncogene and Wnt/β‐catenin signaling in cervical carcinoma cell lines.^37^ Although the description of E7 and Wnt/β‐catenin signaling is not as detailed as E6, the co‐expression of HPV16‐E7 and β‐catenin accelerates the occurrence of cervical carcinoma in a double transgenic mouse model.^38^ In vivo and in vitro experiments have demonstrated that Nr‐CWS can reduce the expression levels of E6 and E7 oncoproteins in cervical carcinoma cells and tumor tissues. The tumor suppressor gene TP53 is involved in tumor necrosis factor‐α (TNF‐α)‐mediated apoptosis by inducing cell cycle arrest, inhibiting cell proliferation, and ultimately leading to apoptosis.^39^ In our experiments, an increase in p53 was also detected at both the transcriptional and translational levels. The above results once again confirmed the inhibitory effect of Nr‐CWS on cervical carcinoma cells. In the cell cycle detection, we found that more cells were blocked in G2/M phase after the addition of Nr‐CWS. In the G2 phase, cells repair DNA damage or incomplete chromosomal replication. The cells prevent further replication of damaged chromosomes by delaying G2/M transformation. p53 also affects the long‐term arrest of G2/M phase, and directly inhibit the expression of cyclin B1‐related genes through transcriptional products. Meanwhile, three downstream genes p21, GADD45, and 14–3‐3σ, of p53 are also involved in G2/M arrest.^40^

The ultimate effect of Nr‐CWS on cervical carcinoma cells is to promote the apoptosis of cervical carcinoma cells. In vivo, we found that the weight and volume of tumors in mice treated with Nr‐CWS were significantly smaller than those in the PBS group. P16, Ki67, and PCNA are indicators for evaluating the malignant degree of tumors, and their expression was consistently decreased.^41^ TNFR1 binds to the junction protein FADD in the cytoplasm, which contains a death domain. When the death domain binds to caspase‐8, the apoptotic protease cascade is turned on. Caspase‐8 cleaves downstream substrates, such as caspase‐3, and interacts with the Bcl‐2 family to initiate apoptosis. The release of apoptotic molecules activates caspase‐9 in the cytoplasm and amplifies the apoptotic protein cascade.^42^ In the in vitro experiments, we found that the apoptosis rate of cervical carcinoma cell lines in the Nr‐CWS group was increased. Expression of Bcl‐2 decreased, while expression of BAX, caspase‐3, caspase‐8, and caspase‐9 increased. We co‐cultured cervical carcinoma cell lines after knocking down TNFR1 with macrophages in the Nr‐CWS environment. After knocking down TNFR1, we found that the effect of Nr‐CWS in promoting apoptosis of cervical carcinoma cells disappeared by enhancing the secretion of TNF‐ɑ by macrophages.

Immunotherapy and chemoradiotherapy are routine treatments for advanced cervical carcinoma. Immunocheckpoint inhibitors, such as the PD‐1 antibody pembrolizumab, are the most common immunotherapy. However, many advanced patients, especially those with low‐PD‐1/PD‐L1 expression, are not sensitive to immunocheckpoint inhibitor therapy.^43^ Our study provides the possibility for the combination of Nr‐CWS with PD‐1/PD‐L1 antibody. A large number of studies have shown that radiation therapy can affect the tumor microenvironment, resulting in increased PD‐L1 side effects in tumor tissues.^44^ In our study, Nr‐CWS treatment reduced PD‐L1 in tumor tissues. This characteristic suggests that Nr‐CWS can be used to resist the side effects of radiation therapy, bringing new hints for the treatment of advanced cervical carcinoma.

This paper only studied the effect of Nr‐CWS on the antitumor function of CD68+ Mφs, which is limited. Differentiated M1 macrophages and M2 macrophages still exist in TME. However, their effects on tumor progression are reversed. M1 macrophages have antitumor and pro‐inflammatory phenotypes and M2 macrophages promote tumor progression and suppress immunity. In our next study, we will conduct an in‐depth study on whether Nr‐CWS can affect the polarization of CD68+ Mφs and the mutual transformation between M1 macrophages and M2 macrophages. One shortcoming of this study is that adherent monocytes remained despite the removal of as many unadherent lymphocytes as possible during culture and induction. T cells, B cells, and other lymphocytes could not be avoided in the process of culture. Because CD80 is also expressed on T cells and B cells and CD68 is also present on monocytes. In future studies, we will add more inducers and identification markers to obtain specific Mφs and DCs. For example, the addition of IFN‐γ and LPS induces M1, and the addition of IL‐4 and IL‐13 induces M2. At the same time, CD86 can be added to mark M1, CD206, and CD163 can be added to mark M2, CD14, and CD86 can be added to mark DCs. We know that Nr‐CWS inhibits the development of cervical carcinoma by enhancing the antitumor effects of dendritic cells and macrophages. However, the underlying mechanism is still being examined. In this paper, the effects of two different concentrations of Nr‐CWS on cervical carcinoma were studied. Experimental results demonstrated that different concentrations have an effect in some aspects, but have no effect in other aspects. Whether the above differences are caused by artifact is still an open question. In future studies, we will try to clarify the mechanism by which Nr‐CWS promotes apoptosis of cervical carcinoma cells by enhancing the antitumor effect of dendritic cells and macrophages, and whether the concentration difference of Nr‐CWS affects its inhibitory effect.

## ETHICS STATEMENT

The studies involving human participants were reviewed and approved by the Ethics Committee of Shengjing Hospital of China Medical University (Shenyang, China). The patients/participants provided their written informed consent to participate in this study. The animal study was reviewed and approved by Ethics Committee of China Medical University (Shenyang, China).

## CONFLICT OF INTEREST

The authors declare that they have no conflict of interest.

## AUTHOR CONTRIBUTIONS

SYZ, MW, and YSL designed the project. MW and YYZ supervised the project. SYZ, HW, and ZZ performed the experiments. SYZ and HW analyzed the data and jointly wrote the manuscript. All authors contributed to the article and approved the submitted version.

## Supporting information

Figure S1

## Data Availability

The datasets used and/or analyzed during the current study are available upon reasonable request.

## References

[1] Allouch S , Malki A , Allouch A , et al. High‐risk HPV oncoproteins and PD‐1/PD‐L1 interplay in human cervical cancer: recent evidence and future directions. Front Oncol. 2020;10:914. 32695664 10.3389/fonc.2020.00914 PMC7338567

[2] Chambuso RS , Rebello G , Kaambo E . Personalized human papillomavirus vaccination for persistence of immunity for cervical cancer prevention: a critical review with experts’ opinions. Front Oncol. 2020;10:548. 32391264 10.3389/fonc.2020.00548 PMC7191065

[3] Farrukh H , El‐Sayes N , Mossman K . Mechanisms of PD‐L1 regulation in malignant and virus‐infected cells. Int J Mol Sci. 2021;22(9):4893. 34063096 10.3390/ijms22094893 PMC8124996

[4] Steinman RM . Decisions about dendritic cells: past, present, and future. Annu Rev Immunol. 2012;30:1‐22. 22136168 10.1146/annurev-immunol-100311-102839

[5] Wculek SK , Cueto FJ , Mujal AM , et al. Dendritic cells in cancer immunology and immunotherapy. Nat Rev Immunol. 2020;20(1):7‐24. 31467405 10.1038/s41577-019-0210-z

[6] Ricketts TD , Prieto‐Dominguez N , Gowda PS , et al. Mechanisms of macrophage plasticity in the tumor environment: manipulating activation state to improve outcomes. Front Immunol. 2021;12:642285. 34025653 10.3389/fimmu.2021.642285 PMC8139576

[7] Li L , Yu R , Cai T , et al. Effects of immune cells and cytokines on inflammation and immunosuppression in the tumor microenvironment. Int Immunopharmacol. 2020;88:106939. 33182039 10.1016/j.intimp.2020.106939

[8] Tan Y , Wang M , Zhang Y , et al. Tumor‐associated macrophages: a potential target for cancer therapy. Front Oncol. 2021;11:693517. 34178692 10.3389/fonc.2021.693517 PMC8222665

[9] Chow A , Zhou W , Liu L , et al. Macrophage immunomodulation by breast cancer‐derived exosomes requires Toll‐like receptor 2‐mediated activation of NF‐κB. Sci Rep. 2014;18(4):5750. 10.1038/srep05750 PMC4102923 25034888

[10] Dong W , Sun S , Cao X , et al. Exposure to TNF‐α combined with TGF‐β induces carcinogenesis in vitro via NF‐κB/Twist axis. Oncol Rep. 2017;37(3):1873‐1882. 28098875 10.3892/or.2017.5369

[11] Shibata T , Lieblong BJ , Sasagawa T , et al. The promise of combining cancer vaccine and checkpoint blockade for treating HPV‐related cancer. Cancer Treat Rev. 2019;78:8‐16. 31302573 10.1016/j.ctrv.2019.07.001 PMC6710123

[12] Guo L , Hua K . Cervical cancer: emerging immune landscape and treatment. OncoTargets and Therapy. 2020;13:8037‐8047. 32884290 10.2147/OTT.S264312 PMC7434518

[13] Chen W , Zhang Y , Zhao C , et al. Nocardia rubra cell wall skeleton up‐regulates T cell subsets and inhibits PD‐1/PD‐L1 pathway to promote local immune status of patients with high‐risk human papillomavirus infection and cervical intraepithelial neoplasia. Front Immunol. 2020;11:612547. 33552075 10.3389/fimmu.2020.612547 PMC7856144

[14] Meng Y , Sun J , Wang X , et al. The biological macromolecule Nocardia rubra cell‐wall skeleton as an avenue for cell‐based immunotherapy. J Cell Physiol. 2019;234(9):15342‐15356. 10.1002/jcp.28182 30697721

[15] Tao Y , Wang G , Zhai J , et al. Functional modulation of CD8+ T cell by approved novel immune enhancer: Nocardia rubra Cell‐Wall Skeletons (Nr‐CWS). Int Immunopharmacol. 2020;78:106023. 31881523 10.1016/j.intimp.2019.106023

[16] Wang G , Wu J , Miao M , et al. Nocardia rubra cell‐wall skeleton promotes CD4(+) T cell activation and drives Th1 immune response. Int J Biol Macromol. 2017;101:398‐407. 28315436 10.1016/j.ijbiomac.2017.03.060

[17] Huang C , Tang X , Li S , et al. Immunopotentiator Aikejia improves the therapeutic efficacy of PD‐1/PD‐L1 immunosuppressive pathway in CT26.WT cancer cell. J Cancer. 2019;10(15):3472‐3480. 31293651 10.7150/jca.29672 PMC6603415

[18] Jia Y , Chen X , Sun J . Apremilast ameliorates IL‐1α‐induced dysfunction in epidermal stem cells. Aging. 2021;13(15):19293‐19305. 34375302 10.18632/aging.203265 PMC8386542

[19] Mantegazza AR , Savina A , Vermeulen M , et al. NADPH oxidase controls phagosomal pH and antigen cross‐presentation in human dendritic cells. Blood. 2008;112(12):4712‐4722. 18682599 10.1182/blood-2008-01-134791 PMC2597138

[20] Chen XJ , Han LF , Wu XG , et al. Clinical Significance of CD163+ and CD68+ Tumor‐associated Macrophages in High‐risk HPV‐related Cervical Cancer. J Cancer. 2017;8(18):3868‐3875. 29151975 10.7150/jca.21444 PMC5688941

[21] Kourtidis A , Lu R , Pence LJ , et al. A central role for cadherin signaling in cancer. Exp Cell Res. 2017;358(1):78‐85. 28412244 10.1016/j.yexcr.2017.04.006 PMC5544584

[22] Kovachev SM . A Review on inosine pranobex immunotherapy for cervical HPV‐positive patients. Infect Drug Res. 2021;14:2039‐2049. 10.2147/IDR.S296709 PMC8180272 34103950

[23] Milano S , Dieli M , Millott S , et al. Effect of isoprinosine on IL‐2, IFN‐gamma and IL‐4 production in vivo and in vitro. Int J Immunopharmacol. 1991;13(7):1013‐1018. 1722191 10.1016/0192-0561(91)90055-c

[24] Sliva J , Pantzartzi CN , Votava M . Inosine pranobex: a key player in the game against a wide range of viral infections and non‐infectious diseases. Adv Ther. 2019;36(8):1878‐1905. 31168764 10.1007/s12325-019-00995-6 PMC6822865

[25] Noy R , Pollard JW . Tumor‐associated macrophages: from mechanisms to therapy. Immunity. 2014;41(1):49‐61. 25035953 10.1016/j.immuni.2014.06.010 PMC4137410

[26] Guo F , Kong W , Zhao G , et al. The correlation between tumor‐associated macrophage infiltration and progression in cervical carcinoma. Biosci Rep. 2021;41(5):BSR20203145. 33928349 10.1042/BSR20203145 PMC8493445

[27] Badillo‐Godinez O , Pedroza‐Saavedra A , Valverde‐Garduño V , et al. Induction of therapeutic protection in an HPV16‐associated mouse tumor model through targeting the human papillomavirus‐16 E5 protein to dendritic cells. Front Immunol. 2021;12:593161. 33717073 10.3389/fimmu.2021.593161 PMC7947241

[28] de Boer EC , De Reijke TM , Vos PC , et al. Immunostimulation in the urinary bladder by local application of Nocardia rubra cell‐wall skeletons (Rubratin) and bacillus Calmette‐Guérin as therapy for superficial bladder cancer: a comparative study. Clin Infect Dis 2000;31(Suppl 3):S109‐S114. 11010835 10.1086/314062

[29] Hatscher L , Amon L , Heger L , et al. Inflammasomes in dendritic cells: Friend or foe? Immunol Lett. 2021;234:16‐32. 33848562 10.1016/j.imlet.2021.04.002

[30] Salem ML , Salman S , Barnawi IO . Brief in vitro IL‐12 conditioning of CD8 + T cells for anticancer adoptive T cell therapy. Cancer Immunol Immunother. 2021;70(10):2751‐2759. 33966093 10.1007/s00262-021-02887-7 PMC10992799

[31] Diaz Arguello OA , Haisma HJ . Apoptosis‐inducing TNF superfamily ligands for cancer therapy. Cancers. 2021;13(7):1543. 33801589 10.3390/cancers13071543 PMC8036978

[32] Mahoney KM , Rennert PD , Freeman GJ . Combination cancer immunotherapy and new immunomodulatory targets. Nat Rev Drug Discovery. 2015;14(8):561‐584. 26228759 10.1038/nrd4591

[33] McMellen A , Woodruff ER , Corr BR , et al. Wnt signaling in gynecologic malignancies. Int J Mol Sci. 2020;21(12):4272. 10.3390/ijms21124272 PMC7348953 32560059

[34] Colás‐Algora N , Millán J . How many cadherins do human endothelial cells express? Cell Mol Life Sci. 2019;76(7):1299‐1317. 30552441 10.1007/s00018-018-2991-9 PMC11105309

[35] Ho GY , Bierman R , Beardsley L , et al. Natural history of cervicovaginal papillomavirus infection in young women. N Engl J Med. 1998;338(7):423‐428. 9459645 10.1056/NEJM199802123380703

[36] zur Hausen H . Papillomaviruses and cancer: from basic studies to clinical application. Nat Rev Cancer. 2002;2(5):342‐350. 12044010 10.1038/nrc798

[37] Lichtig H , Gilboa DA , Jackman A , et al. HPV16 E6 augments Wnt signaling in an E6AP‐dependent manner. Virology. 2010;396(1):47‐58. 19896689 10.1016/j.virol.2009.10.011

[38] Bulut G , Fallen S , Beauchamp EM , et al. Beta‐catenin accelerates human papilloma virus type‐16 mediated cervical carcinogenesis in transgenic mice. PLoS One. 2011;6(11):e27243. 22087269 10.1371/journal.pone.0027243 PMC3210148

[39] Yan L , Huang H , Zhang Y , et al. Involvement of p53‐dependent apoptosis signal in antitumor effect of Colchicine on human papilloma virus (HPV)‐positive human cervical cancer cells. Biosci Rep. 2020;40(3):BSR20194065. 32163135 10.1042/BSR20194065 PMC7098170

[40] Merlin JPJ , Rupasinghe HPV , Dellaire G , et al. Role of dietary antioxidants in p53‐mediated cancer chemoprevention and tumor suppression. Oxid Med Cell Longev. 2021;2021:9924328. 34257824 10.1155/2021/9924328 PMC8257365

[41] Kreuter A , Jesse M , Potthoff A , et al. Expression of proliferative biomarkers in anal intraepithelial neoplasia of HIV‐positive men. J Am Acad Dermatol. 2010;63(3):490‐498. 20006407 10.1016/j.jaad.2009.08.043

[42] Yi F , Frazzette N , Cruz AC , et al. Beyond cell death: new functions for TNF family cytokines in autoimmunity and tumor immunotherapy. Trends Mol Med. 2018;24(7):642‐653. 29880309 10.1016/j.molmed.2018.05.004 PMC7466867

[43] Walsh RJ , Tan DSP . The role of immunotherapy in the treatment of advanced cervical cancer: current status and future perspectives. J Clin Med. 2021;10(19):4523. 34640541 10.3390/jcm10194523 PMC8509251

[44] Dyer BA , Feng CH , Eskander R , et al. Current status of clinical trials for cervical and uterine cancer using immunotherapy combined with radiation. Int J Radiat Oncol Biol Phys. 2021;109(2):396‐412. 32942005 10.1016/j.ijrobp.2020.09.016

