# Supplementary figures and images for: Nocardia rubra cell‐wall skeleton influences the development of cervical carcinoma by promoting the antitumor effect of macrophages and dendritic cells
Source: Cancer Med. 2022 Jan 7;11(5):1249–68. doi: 10.1002/cam4.4526 (PMC8894708; doi:10.1002/cam4.4526)

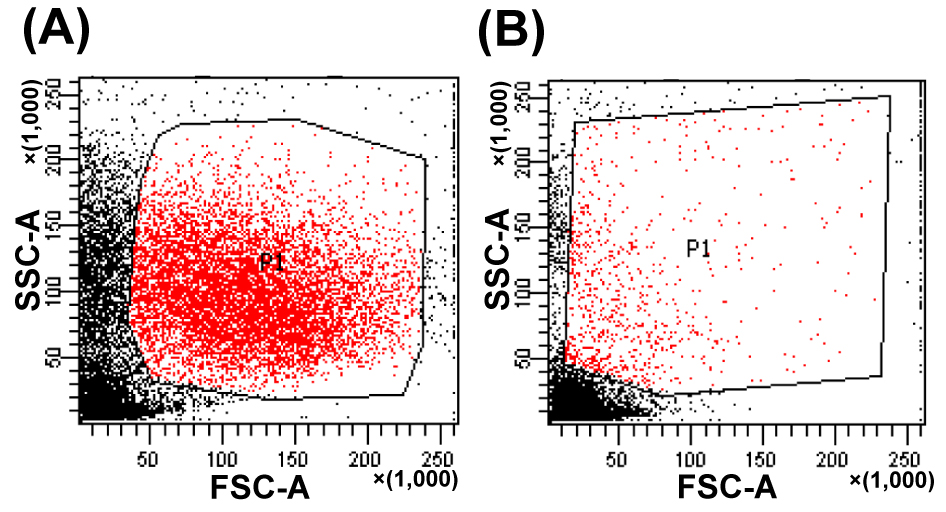

Supplement: Supplementary file 1 — Figure S1 [file CAM4-11-1249-s001.jpg]
